# Prognostic Relevance of Weight and Weight Loss during Multimodal Therapy for Oesophagogastric Tumours

**DOI:** 10.3390/curroncol29040221

**Published:** 2022-04-12

**Authors:** Alessandro Lorusso, Dmitry Bichev, Anica Högner, Prisca Bartels, Alexej Ballhausen, Christoph Treese, Matthias Biebl, Peter Thuss-Patience

**Affiliations:** 1 Department of Hematology, Oncology and Cancer Immunology, Charité—Universitätsmedizin, 13353 Berlin, Germany; anica.hoegner@charite.de (A.H.); prisca.bartels@charite.de (P.B.); alexej.ballhausen@charite.de (A.B.); christoph.treese@charite.de (C.T.); peter.thuss@charite.de (P.T.-P.); 2 Klinikum Barnim GmbH, Werner Forßmann Krankenhaus, 16225 Eberswalde, Germany; dmitry.bichev@klinikum-barnim.de; 3 Department for Medicine (Gastroenterology, Infectious Diseases, Rheumatology), Charité—Universitätsmedizin, Berlin, Corporate Member of Freie Universität Berlin and Humboldt-Universität zu Berlin, 12203 Berlin, Germany; 4 Department of Surgery, Campus Virchow Klinikum, Charité-Universitätsmedizin, 13353 Berlin, Germany; matthias.biebl@charite.de

**Keywords:** gastric cancer, oesophageal adenocarcinoma, multimodal therapy, weight loss, prognosis, survival, neoadjuvant chemotherapy

## Abstract

The prognostic meaning of weight loss (WL) during standard treatment for operable oesophagogastric cancer is still unclear. The aim of this study is to analyse the prognostic effect of WL during perioperative chemotherapy (PC) for gastric cancer (GC) and oesophageal adenocarcinomas (OAC). We retrospectively analysed data from 128 patients (pts) with GC and OAC who underwent surgery in the context of multimodal treatment with PC. We collected data on WL during different steps of therapy together with other histopathologic and demographic information. We analysed the effects on overall survival (OS) and disease-free survival (DFS). Results: Pts with WL ≥ 5% during neoadjuvant chemotherapy exhibited significantly worse OS compared with pts with WL < 5% (median OS: 23.6 months [95% CI: 4.4–42.9] vs. 63.5 months [95% CI: 50.7–76.2], *p* = 0.007) and DFS (median DFS: 12.5 months [95% CI: 2.9–22.1] vs. 63.5 months [95% CI: 31.6–95.4], *p* = 0.016). Pts with WL ≥ 14% during the whole treatment exhibited significantly worse OS compared with pts with WL < 14% (median OS: 43.7 months [95% CI: 13.2–74.2] vs. not reached, *p* = 0.028) and DFS (median DFS: 34.3 months [95% CI: 14.0–54.5] vs. not reached, *p* = 0.038). Conclusion: WL patterns during neoadjuvant chemotherapy and during the whole treatment correlate with a significantly worse prognosis in operated pts with curative GC or OAC in the context of a multimodal treatment with PC. A validation of this prognostic effect in prospective studies is warranted.

## 1. Introduction

Cancers of the upper gastrointestinal tract (upper-GI) represent one of the most common malignancies worldwide [1,2]. According to the last GLOBOCAN survey, gastric adenocarcinoma is the sixth most common tumour in incidence and the fourth most common tumour for tumour-related death [1,2]. Although less frequent (ninth for frequency), oesophageal cancer is the sixth leading cause of cancer death [1]. These cancers also have high symptom burdens and a huge impact on quality of life (QoL) [2]. Adenocarcinomas of the lower oesophagus (OAC) and gastric adenocarcinomas (GC) are generally treated in a similar way because of their biological and clinical similarities [3]. In western countries these entities are treated with a multimodal concept consisting of perioperative chemotherapy [4,5,6,7]. One of the most frequent symptoms of tumour patients (pts), especially those suffering from upper-GI tumours, is weight loss (WL) [8]. Although it is an nonspecific symptom of many malignancies, WL has been at a higher incidence in pts with tumours of the upper-GI for decades [9,10]. The genesis of WL in these pts is multifactorial. Tumour-associated dysphagia, tumour wasting syndrome, and metabolic changes are all shown to play an important role [11,12,13]. WL was shown to affect prognosis in cancer populations, but generally those studies were performed in heterogeneous populations of pts with different entities, different stages, and different therapeutical approaches (e.g., palliative vs. curative) [9,10,14]. The prognostic effect of weight and WL in these pts, especially in the ones with curative options, is therefore far from being fully understood due to these limitations. To address this question, we retrospectively analysed the prognostic value of weight and weight loss in 128 pts with adenocarcinoma of the stomach or of the oesophagogastric junction during curative multimodal therapy.

## 2. Materials and Methods

**Figure 1:**
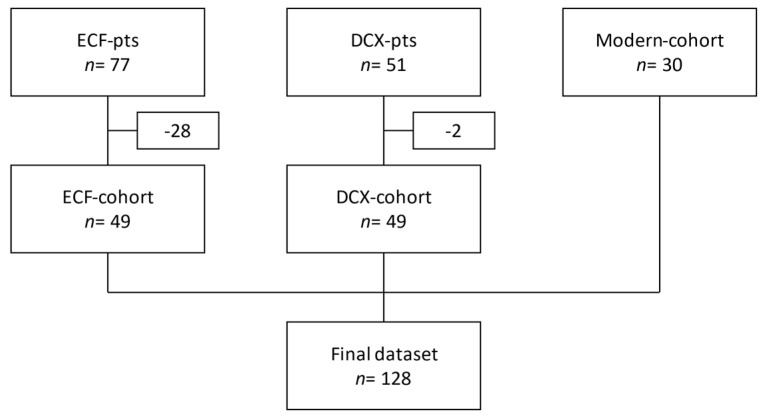
Schematic representation of dataset construction process.

We retrospectively enrolled 128 pts in our analysis that underwent surgery for upper-GI tumours in the context of a multimodal therapy (perioperative chemotherapy) between January 2005 and November 2018. We analysed the weight of the pts at 4 different time points: at diagnosis (i.e., before neoadjuvant therapy), before surgery (i.e., after neoadjuvant therapy), after surgery, and after adjuvant therapy or, in case no adjuvant therapy was performed, 3 months (±2 weeks) after surgery. Inclusion criteria were histological diagnosis of GC or OAC, curative multimodal therapy, perioperative chemotherapy, absence of distant metastases, application of at least one cycle of neoadjuvant chemotherapy, surgery performed, and availability of weight information for at least 2 of the 4 planned time points (one of them being the weight at diagnosis). Exclusion criteria were use of radiotherapy, histological diagnosis of squamous cell carcinomas, palliative therapy, or missing weight information. The final cohort resulted from merging pts from 3 different cohorts (Figure 1).

The first cohort (“ECF-cohort”, *n* = 49) was based on pts who received their curative treatment between 2005 and 2008 as part of the ECF study [15]. This was a monocentric study performed at our institution, in which pts with upper-GI tumours received ECF (epirubicin/cisplatin/5-FU) in the context of multimodal therapy. From the initial 77 pts, we excluded 28 pts who were missing reliable weight information or who did not undergo surgery. The second cohort (“DCX-cohort”, *n* = 49) was based on pts treated in the DCX study between 2008 and 2010. This was a multicentre study in which pts received docetaxel/cisplatin/capecitabine as neoadjuvant and adjuvant therapy as the chemotherapeutic backbone in a curative multimodal therapy [16]. In this case, we excluded 2 pts from the initial 51 pts for missing weight information or not undergoing surgery. Lastly, we also enrolled 30 pts (“modern-cohort”) treated at our institution between 2016 and 2018 who fulfilled inclusion and exclusion criteria with complete datasets available. We retrospectively collected the following data from pts in the final cohort: demographics (age, sex), height, weight at the above listed time points, number of cycles and type of neoadjuvant and adjuvant chemotherapy, histological classification of the tumour entities according to Laurén and WHO classification, tumour localisation, and TNM stage. Since the pts were diagnosed over a long period of time in which 3 different UICC-classifications were used, we retrospectively analysed the staging diagnostics and reclassified all pts according to the eighth edition of the TNM [17]. We further analysed the pathological regression stages according to Becker [18,19] and dates of diagnosis, surgery, progression, death as of its cause, and of concurring postoperative complications. We calculated the BMI according to the WHO formula [20] and weight loss between the different time points as percentage of the weight at diagnosis. We performed the statistical analysis with IBM SPSS 25. Student t-, chi-square, and Mann–Whitney U tests were used to compare descriptive parameters in different pts groups. Kaplan–Meier curves were used to calculate overall survival (OS) and disease-free survival (DFS). OS was defined as the time between diagnosis and death or last follow-up. DFS was defined as the time between diagnosis and relapse, progression, or death. A linear logistic regression was used for further multivariate analysis. After identifying statistically significant variables in the cohort, we then performed an explorative Cox regression analysis to try to investigate their impact on pts’ survival.

## 3. Results

### 3.1. Demographics

Pts and tumour characteristics are summarised in Table 1. In short, median age at diagnosis was 64 years (29–83). There was a predominance of male pts (*n* = 108, 84.4%). Both tumour localisations were almost equally represented in the cohort, with 68 OAC and 60 GC (inclusive AEG-III). According to the Laurén classification, intestinal and diffuse types were diagnosed in 57 pts (44.5%) and 41 pts (32%), respectively. Signet ring cell histology according to the WHO classification was seen in 38 pts (29.7%). All pts were M0 and 15 pts (11.7%) were N0. According to the 8th UICC TNM Classification [17] 66.2% of the pts (*n* = 45) with OAC were in stage 3 and 32.4% (*n* = 22) in stage 4a. For GC, most of the pts (*n* = 46, 76.7%) were in stage 3. Gender-related frequencies (male population only) are summarised in the Supplementary Materials (Table S1).

### 3.2. Chemotherapy

At least one cycle of neoadjuvant chemotherapy was applied in every patient, with 89.1% (*n* = 114) of the pts receiving three (*n* = 90) or four (*n* = 24) cycles. The most frequently applied neoadjuvant chemotherapies were DCX (*n* = 49, 38.3%), ECF (*n* = 45, 35.2%), and FLOT (*n* = 21, 16.4%). Other combinations were rare. As expected, only a portion of the pts (*n* = 81, 63.3%) were able to receive postoperative adjuvant chemotherapy. Of the 47 pts (36.7%) who were not able to receive adjuvant chemotherapy, the reasons were as follows: 9 pts (19.1%) because of progressive disease after surgery, 26 (55.3%) because of low performance status, 8 (17%) because of patient refusal, and for 4 pts (8.5%) the cause was not known. Of the 81 pts who received postoperative chemotherapy, 48 (59.2%) received three cycles, 15 (18.5%) received four cycles, and 12 and 7 pts (14.8% and 8.6%) received two cycles and one cycle, respectively. DCX was applied in 36 pts (44.4%), ECF in 20 (24.7%), and FLOT in 11 (13.6%). Other combinations were rare. Results of neoadjuvant and adjuvant chemotherapy are summarised in Table 2 and Table 3. Gender-related frequencies (male population only) are summarised in the Supplementary Materials (Tables S2 and S3).

### 3.3. Weight and Weight Loss

**Figure 2:**
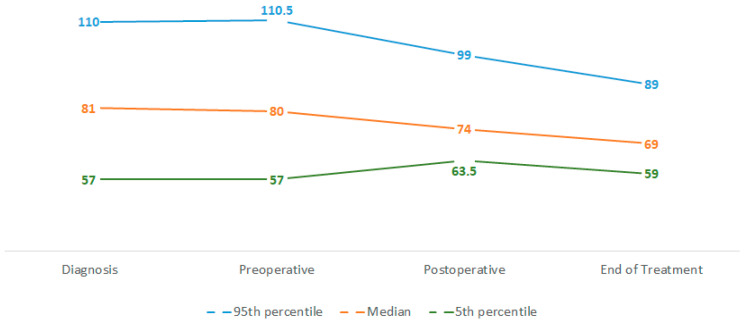
Weight dynamics in the cohort during observation. Values expressed in kilograms (median, 5th, and 95th percentile).

We collected information about weight at four different time points throughout the treatment. The phases between these time points were the object of our analysis, and we focused on the “neoadjuvant phase” (between diagnosis and surgery, analysable *n* = 128), the “adjuvant phase” (between surgery and end of the treatment, analysable *n* = 98), and the “whole treatment phase” (between diagnosis and end of treatment, analysable *n* = 104). We then calculated WL as percentage decrease of weight compared to weight at time of diagnosis. During neoadjuvant chemotherapy we found a median WL of 0%, meaning weight stability (range: WL 23% to weight gain [WG] 16%). Twelve pts (9.4%) had a WL of at least 5% and four pts (3.1%) lost at least 10% of their initial weight. Forty pts (31.2%) gained at least 1% of their initial weight in this phase. In the adjuvant phase, we found a median WL of 4% (range: WL 24%—WG 8%) in the 98 (76.6%) analysable pts. The other 30 pts were not analysable because of missing information about postoperative weight (*n* = 6), weight at the end of the treatment (*n* = 17), or both (*n* = 7). A median body WL of 14% was seen during the whole treatment (range: WL 33%–WG 7%). Of the 104 analysable pts, only 2 pts did not lose weight: one remained stable, one had a weight gain of 7%, whereas 93 pts (89.4%) had at least a 5% WL and 76 (73.1%) had at least a 10% WL in this phase. Weight loss dynamics are summarised in Figure 2 and Table 4.

### 3.4. Surgical Aspects

All pts underwent surgery, with 53 pts (41.4%) receiving a total gastrectomy, 45 pts (35.2%) an abdominothoracic oesophagectomy, 22 pts (17.2%) a transhiatal extended gastrectomy, 6 pts (4.7%) a partial gastrectomy, and 2 pts (1.6%) a Whipple procedure. Every patient received a D2 lymphadenectomy. The analysis of Becker regression status showed a complete response (Becker 1a) in 20 pts (15.6%), a subtotal remission (Becker 1b) in 11 pts (8.6%); a partial remission (Becker 2) was shown in 33 pts (25.8%) and no remission (Becker 3) was shown in 63 pts (49.2%). For one pt (0.7%) the information about regression status was missing, and 54 pts (42.2%) experienced postoperative complications, mainly infections (*n* = 26, 48.1%) or anastomotic leakage (*n* = 8; 14.8%).

### 3.5. Survival

Median follow-up was 49.7 months (95% CI: 43.4–56). There were 55 (42.9%) deaths, 37 of the 55 pts (67.3%) died of tumour progression, whereas 14 pts (25.5%) died of other causes. One pt (1.8%) died after surgery, for three pts (5.4%) the cause of death was not known. Median overall survival (OS) was 60.8 months (95% CI:37.4–84.2). Median disease-free survival (DFS) was 60.8 months (95% CI: 35.2–86.5) (Figure S1, Supplementary Materials).

**Figure 3:**
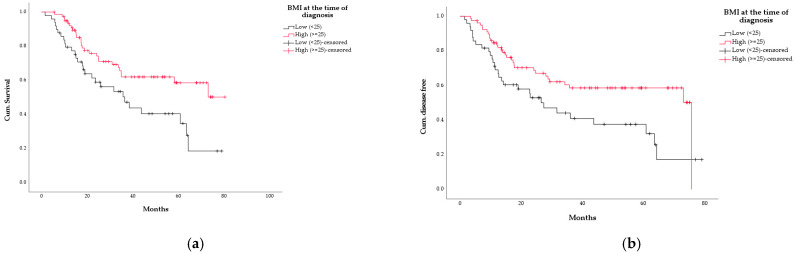

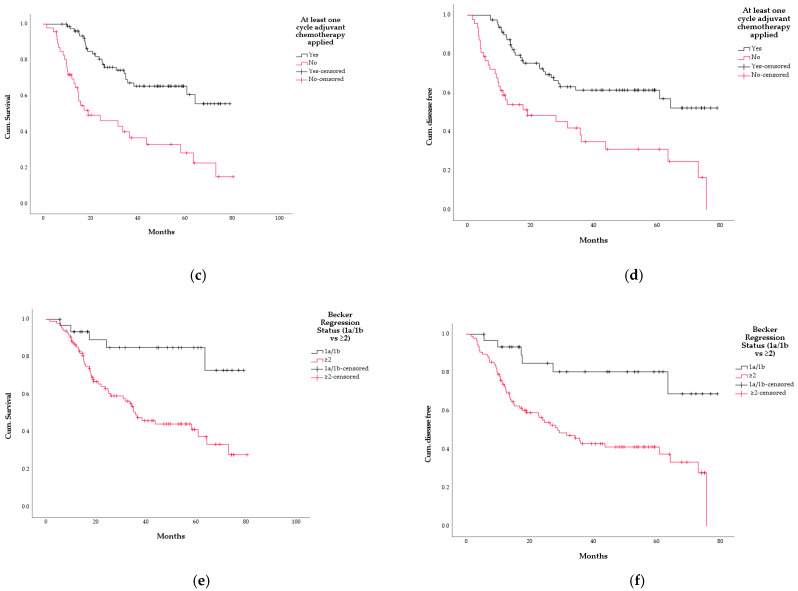
Kaplan–Meier curves according to different variables, as listed: (**a**) overall survival according to BMI (red line = BMI ≥ 25 kg/m2, *n* = 79; black line = BMI ≥ 25 kg/m2, *n* = 49; *p* = 0.010); (**b**) disease-free survival according to BMI (red line: BMI ≥ 25 kg/m2, *n* = 79; black line = BMI ≥ 25 kg/m2, *n* = 49; *p* = 0.013); (**c**) overall survival according to application of adjuvant chemotherapy (black line = at least 1 cycle, *n* = 81; red line = no adjuvant chemotherapy, *n* = 47; *p* = 0.000); (**d**) disease-free survival according to application of adjuvant chemotherapy (black line = at least 1 cycle, *n* = 81; red line = no adjuvant chemotherapy, *n* = 47; *p* = 0.000); (**e**) overall survival according to Becker regression status, 127 analysable pts (black line = Becker regression status 1a/1b, *n* = 31; red line = Becker regression status ≥ 2, *n* = 96; *p* = 0.001); (**f**) disease-free survival according to Becker regression status, 127 analysable pts (black line = Becker regression status 1a/1b, *n* = 31; red line = Becker regression status ≥ 2, *n* = 96; *p* = 0.001).

Pts with a BMI at the time of diagnosis of at least 25 kg/m2 had a significant survival benefit (*p* = 0.01), without reaching median OS, whereas pts with BMI <25 kg/m2 had a median OS of 36.5 months (95% CI 20.9–52). Pts with a BMI at diagnosis of ≥25 kg/m2 also had a DFS benefit with a median DFS of 75.6 months (95% CI: 35.1–86.4) versus a DFS of 26.5 months (95% CI: 14.8–38.2) for the pts with a BMI less than 25 kg/m2 (*p* = 0.013) (Figure 3a,b).

Pts who received adjuvant chemotherapy also had a survival benefit compared to pts who did not (median OS not reached vs. 19 months [95% CI: 1–36.9], *p* = 0.000; median DFS not reached vs.19 months [95% CI: 0–39.3], *p* = 0.000) (Figure 3c,d).

Regression status according to Becker also showed an effect on survival, where pts with Becker 1a and 1b did not reach median OS, and pts with Becker ≥2 had a median OS of 35.7 months (95% CI: 24.8–46.7; *p* = 0.001). DFS was also affected, with median DFS not reached for pts with Becker 1a/1b versus a median DFS of 28.7 months (95% CI: 17.4–40.1) for the pts with Becker ≥2 (*p* = 0,001) (Figure 3e,f).

Tumour localisation (OA vs. GC), postoperative complications, or histology had no effect on survival (data not shown).

We then analysed the effect of weight loss patterns on survival. We chose the WL thresholds for our analysis according to the median WL for each phase observed in our cohort. Since median WL of the neoadjuvant phase was 0%, we chose in this particular phase a different threshold, based on published data [8,9,10,21], that could better represent the pathological nature of WL. Thus, we analysed the pts according to their weight loss during the whole treatment (≥14%, i.e., median WL), during the neoadjuvant phase (WL ≥5%, thresholds based on published data [8,9,10,21]), and during the adjuvant phase (≥4%, i.e., median WL).

**Figure 4:**
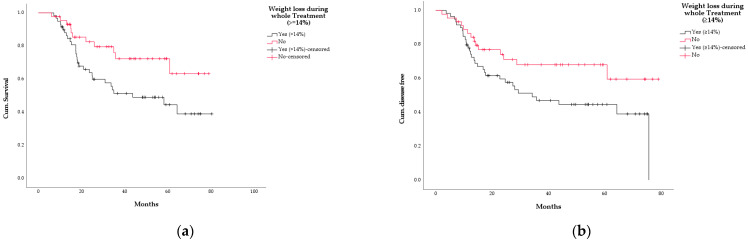
Kaplan–Meier curves according to weight loss during the whole treatment (threshold: WL ≥ 14%, 104 analysable pts). (**a**) overall survival (black line = WL ≥ 14%, *n* = 59; red line = WL < 14%, *n* = 45; *p* = 0.028); (**b**) disease-free survival (black line = WL ≥ 14%, *n* = 59; red line = WL < 14%, *n* = 45; *p* = 0.038).

For the whole treatment phase (104 pts analysable), we could find that pts who lost at least 14% of their initial weight (*n* = 59, 56.8%; median OS not reached) during the whole treatment had a worse survival compared to pts who did not (*n* = 45, 43.2%, OS 43.7 months, 95% CI: 13.2–74.2, *p* = 0.028), as well as a worse DFS (not reached vs. 34.3 months, 95% CI: 14–54.5, *p* = 0.038) (Figure 4).

**Figure 5:**
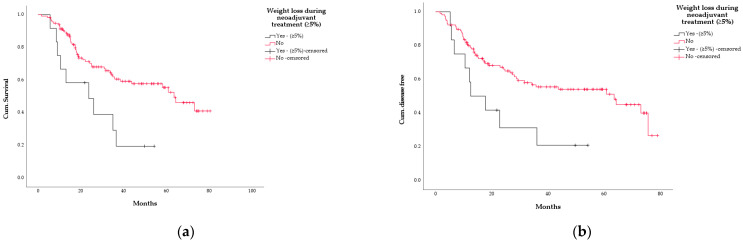
Kaplan–Meier curves according to weight loss during neoadjuvant therapy (threshold: WL ≥ 5%, 128 analysable pts). (**a**) overall survival (black line = WL ≥ 5%, *n* = 12; red line = WL < 5%, *n* = 116 *p* = 0.007); (**b**) disease-free survival (black line = WL ≥ 5%, *n* = 12; red line = WL<5%, *n* = 116; *p* = 0.016).

Analysing weight change during the neoadjuvant phase, the 12 pts (9.3%) with a WL of at least 5% during the neoadjuvant phase showed a worse OS compared with the ones with WL < 5% or with weight gain (23.6 months [95% CI: 4.4–42.9] vs.63.5 [95% CI: 50.7–76.2], *p* = 0.007) as well as a significantly worse DFS (12.5 months [95% CI: 2.9–22.1] vs. 63.5 months [95% CI: 31.6–95.4], *p* = 0.016) (Figure 5).

Weight loss patterns during the adjuvant phase (analysed at different thresholds: ≥0%; ≥4%, i.e., median WL; ≥5%; or ≥10%) showed no association with either OS and DFS (data not shown). TNM-stage correlated with survival according to already published data. Since this analysis was not the focus of our study, the results are not shown.

All gender-related survival curves (male population only) are shown in the Supplementary Materials (Figures S2–S5).

### 3.6. Association between WL and Other Variables

We performed a crosstab to analyse the possible correlation between WL patterns and other relevant variables. A WL of ≥5% in the neoadjuvant phase and ≥14% during the whole treatment showed no correlation with Becker regression status, nor with the feasibility of adjuvant chemotherapy, nor with the frequency of postoperative complications. In the Mann–Whitney analysis no effect of T or N stage on WL patterns was shown. When explored in a multivariate analysis, N status, application of adjuvant chemotherapy and Becker regression status had no association with a WL ≥ 5% in the neoadjuvant phase or with WL ≥ 14% during the whole treatment.

### 3.7. Cox Regression Analysis

To investigate the impact of the variables on OS and DFS, we performed an explorative Cox regression analysis with following covariates: BMI at time of diagnosis (<25 kg/m2 vs. ≥ 25 kg/m2), Becker regression status (1a/1b vs. ≥2), application of adjuvant chemotherapy (at least 1 cycle applied vs. not applied), WL ≥ 5% during neoadjuvant chemotherapy (yes vs. no), and WL ≥ 14% during the whole treatment (yes vs. no).

The Cox regression analysis for OS and DFS confirmed the positive prognostic value of a Becker regression status of 1a/1b as well as of the application of adjuvant chemotherapy. A BMI < 25 kg/m2 at time of diagnosis was not significantly associated with worse OS. A WL of at least 5% during neoadjuvant therapy and of 14% during the whole treatment also did not reach statistical significance. Results are summarised in Table 5 and Table 6.

## 4. Discussion

Our retrospective analysis was focused on a better comprehension of the prognostic effect of weight loss dynamics during treatment in a homogeneous, curative GC and OAC patient population under multimodal treatment with perioperative chemotherapy and surgery.

Weight loss prior to diagnosis has been shown to have an impact on survival in many oncological pts, and GC and OAC are not excluded. A Japanese retrospective study on 1330 pts with curative GC showed that a WL before surgery has negative prognostic impact on survival (HR: 1.152, 95% CI: 1.014–1.310, P = 0.030) [21]. According to the Japanese guidelines, these pts were treated without prior neoadjuvant chemotherapy or with an adjuvant chemotherapy if indicated. This finding strongly supports that WL is not only a symptom but also a clinical sign with a measurable effect on survival for pts with GC at diagnosis.

Up to 85% of pts with GC or OAC present at diagnosis with WL. Studies show a median WL prior to diagnosis from 5% up to 15% depending on the disease status and nearly half of the pts have WL of at least 10% [8,9,10]. The genesis of WL in upper-GI tumour pts is multifactorial. On one hand, pts with tumours have a reduced intake of nutrients because of tumour-associated or therapy-associated symptoms like dysphagia, nausea, or anorexia. On the other hand, reduced patient activity promotes muscle-wasting processes. Finally, the tumour itself activates through cytokines different pathways leading to a systemic “catabolic phenotype” in the host [11,12,13].

Our study shows that pts experienced a beneficial effect on weight during neoadjuvant chemotherapy, with a weight stabilisation (WL ≤ 0%) in almost 50% and a weight gain (WL < 0%) in almost 30% of the cohort during the first part of the therapy, with only a small percentage (c. 10%) of pts suffering further significant weight loss (≥5% of initial weight).

The positive effect of neoadjuvant therapy seen in our study is probably multifactorial. Supportive therapy like parenteral or supplemental oral nutrition as well as the use of stents in stenotic tumours could help the patient stop losing weight. This information was unfortunately not available in our cohort and could not be analysed.

On the other hand, the effectiveness of chemotherapy may also play a determinant role. Reduction of tumour burden and amelioration of dysphagia due to tumour shrinking also improve alimentation and QoL in these pts. Finally, a reduction in tumour-associated inflammation positively impacted the patients’ metabolic status. Our study is one of the first investigating WL during neoadjuvant chemotherapy.

For the palliative situation, the prognostic relevance of WL has been investigated: Ock et al. showed in 719 pts with metastatic GC that a WL of 3% in the first month of palliative chemotherapy was associated with a worse survival (8.9 vs. 15.3 months, HR 0.66) [22]. The explanation of this finding is that weight change could be a surrogate parameter of chemotherapy efficacy, at least in palliative situations.

Similar results have been recently published also in other entities. In a retrospective analysis in pts with unresectable colorectal cancer undergoing first-line palliative therapy with FOLFIRI and cetuximab or bevacizumab (FIRE-3 Study) a WL of ≥5% at 3 months was predictive of worse survival (32.4 vs. 21.1 months; hazard ratio [HR]: 1.64; 95% CI: 1.13–2.38; *p* = 0.0098) and progression-free survival (11.8 vs. 9.0 months; HR: 1.72; 95% CI: = 1.18–2.5; *p* = 0.0048) [23]. This also supports the evidence of WL dynamics as surrogate parameters of chemotherapy efficacy since dysphagia-associated WL plays a minor role in distal colorectal cancer compared with GC or OAC.

Although in our study no association between WL patterns and pathological response was seen, a retrospective study of 203 GC pts from Jiang et al. showed that, together with histology and age, WL during neoadjuvant chemotherapy was a predictive factor for worse pathological response [24]. An analysis on survival was not performed.

To our knowledge, no studies until now have evaluated the role of WL dynamics during therapy on survival in curative GC and OAC pts, since most studies are performed in heterogeneous or palliative populations. We found that a WL of at least 5% during neoadjuvant therapy was associated with a worse OS and DFS. Taken together with the above mentioned findings, it is legitimate to hypothesise that neoadjuvant chemotherapy could disrupt the negative prognostic effect of WL in most GC and OAC pts through its antitumour efficacy, while the ones further losing weight during treatment would still show a worse survival.

We were also able to find that a WL of at least 14% during the whole treatment was associated with both a worse OS and DFS. Pts with reduced responses to therapy may experience not only persistent dysphagia but also the persistence of micrometastases, inflammation, and frequent complications during therapy or after surgery, which further promotes WL, eventually worsening survival. Additionally, pts experiencing WL could have limited access to postoperative therapy and/or tolerate them worse [25,26]. These factors could lead to a negative effect on prognosis. In our study, persisting WL during the whole treatment was associated with worse survival. A hypothesis would be that more aggressive disease, with a tendency to micrometastatic persistence after treatment in the neoadjuvant and operative phase, could cause further WL even after “macroscopic” cure of the patient. Exploring the pathophysiological pathways that can cause this WL pattern during the whole treatment to affect survival is beyond the scope of this study. Nonetheless, this interesting hypothesis will hopefully be addressed in future studies in prospective cohorts.

This study has some limitations. First, the retrospective nature of the study is a limitation. This aspect affected data availability, with some pts missing reliable weight information. In addition, we enrolled only pts who underwent surgery, thus excluding the ones who experienced progressive disease, died, or became too unfit for surgery during neoadjuvant therapy. Even though these pts were, numerically speaking, a small minority, they represent a group with poor prognosis that was excluded from final analysis, possibly affecting statistical results. This bias towards selection of pts with a better prognosis is indeed visible in the OS and DFS of the whole cohort, which are superior to the expected results if compared with published data [27]. Unfortunately, data about supportive therapies (e.g., nutritional support) are missing. It would have been ideal to correlate nutritional support with weight changes, but as all pts were treated in our institution under the same local guidelines for nutritional support as needed, we assumed that these data would not change our results. Similarly, information about the presence of WL prior to diagnosis is missing and only a comparison with published data is possible.

Secondly, despite our effort to collect a homogenous cohort of pts, differences in the chemotherapy due to the wide time window in which the pts were retrospectively enrolled may have affected results.

Despite the relatively small number of pts in our cohort we performed an exploratory Cox regression analysis to evaluate the impact of the variables of interest on survival. The interpretation of these results should be done with great caution since the small numbers of the cohort could negatively affect the power of the analysis. The WL patterns explored in our analysis did not reach statistical significance, even though showing a trend (especially for WL ≥ 5% in OS, *p* = 0,075). Many points could explain this result. First, the pts with a WL of at least 5% during the neoadjuvant phase represent a minority of our cohort. In a larger cohort, statistical significance may have been reached. Secondly, the effect of WL patterns on survival may not have been as strong as the effect of other concurring variables. Finally, WL is an easy tool to evaluate and summarise body composition dynamics in these pts but at the same time could be affected by other disturbing events (e. g., fluid retention), hampering its descriptive power. Future studies with more reliable measurement are warranted (see below).

Despite such limitations, our study confirms the hypothesis of the prognostic effect of WL in this subset of pts. In our analysis prognostic relevant WL patterns were not affected by other variables such as TNM, postoperative complications, histology, or Becker regression status.

Our data about the prognostic effect of WL in curative GC and OAC pts need further confirmation in larger, prospective cohorts before their use could guide everyday practice in identifying pts who could benefit from personalised supportive, oncological, or surgical therapies to overcome this negative effect.

## 5. Future Perspectives

The present study suggests a prognostic effect of WL in a relatively homogenous cohort of curative GC and OAC pts. Although the use of a simple tool such as weight in defining groups with worse prognosis could be fascinating, it is important to underline how weight itself may not be as reliable as other parameters in summing up the real nutritional and inflammatory status of pts since it can be rapidly modified by fluid balance and does not summarise the real body composition of the pts (fat tissue and muscle mass). Promising is the use the evaluation of sarcopenia (intended as loss of lean muscle mass) and/or the quantification of visceral fat, for example, through CT morphological criteria, to better evaluate the real nutritional, metabolic, and inflammatory status of the patient. Koch et al. showed in a subgroup analysis of 83 pts with GC or OAC of the FLOT4-Trial that the presence of CT-defined sarcopenia at diagnosis affects survival [28]. Unfortunately, no dynamic evaluation throughout therapy was performed, leaving open the question of whether changes during therapy could also identify subgroups of pts with different prognoses. The role of inflammation in nutritional status and prognosis could be clearly seen in different studies analysing the prognostic role of NLR (neutrophil to lymphocyte ratio [29,30]) or PNI (prognostic nutritional index [31,32]) in pts with GC or OAC. Future studies should aim for the integration of clinical and CT-based sarcopenia evaluation and biochemical analysis of inflammatory status together with weight to better explain how cancer and oncological treatment elicit their effect on WL, clarifying whether a specific therapy for these processes is possible.

## 6. Conclusions

Neoadjuvant therapy has a beneficial effect on WL in most pts with curative GC and OAC. WL patterns during neoadjuvant therapy (≥5%) and during the whole treatment (≥14%) in pts with GC and OAC undergoing multimodal therapy can identify a subgroup of pts with worse survival. Further studies should help to explore more precise instruments to identify such pts as well as possible individualised therapeutical measures to overcome this negative prognostic effect.

## Figures and Tables

**Table 1:** Patient characteristics.

Variable *n* = 128	Median	Range
Age	64	29–83
Weight	81 kg	45–118 kg
BMI	25.7 kg/m^2^	16.5–39.4 kg/m^2^
	Frequency	Percentage
Sex		
Females	20	15.6%
Males	108	84.4%
Localisation		
Oesophagus	68	53.1%
Stomach (incl. AEG III)	60	46.9%
Histology		
Adenocarcinoma	128	100%
Laurén Histology		
Intestinal Type	57	44.5%
Diffuse Type	41	32%
Mixed Type	10	7.8%
Not specified	20	15.6%
WHO Histology		
Papillary	7	5.5%
Mucinous	6	4.7%
Tubular	26	20.3%
Signet Ring Cell	38	29.7%
Undifferentiated	10	7.8%
Not specified	41	32%

**Table 2:** Characteristics of neoadjuvant and adjuvant chemotherapy. Sum of percentages could be ≠100% because of rounding error.

Chemotherapy Performed	Frequency	Percentage
Neoadjuvant	128	100%
Adjuvant	81	63.3%
Nr. of cycles (neoadjuvant)		
≤2	11	8.6%
3–4	114	89.1%
≥5	3	2.3%
No. of cycles (adjuvant)		
none	47	36.7%
≤2	19	14.8%
≥3	62	48.4%

**Table 3:** List of the most used chemotherapy regimens. Sum of percentages could be ≠100% because of rounding error.

Regimens ^1^	Frequency	Percentage
Neoadjuvant (*n* = 128)		
DCX	49	38.3%
ECF	45	35.2%
FLOT	21	16.4%
FLOT-like	8	6.2%
ECF-like	5	3.9%
Adjuvant (*n* = 81)		
DCX	36	44,4%
ECF	20	24.7%
FLOT	11	13.6%
FLOT-like	9	11.1%
ECF-like	4	4.9%
Others	1	1.2%

^1^ DCX = docetaxel, cisplatin, capecitabin; ECF = epirubicin, cisplatin, 5-FU; FLOT = 5-FU, leukovorin, oxaliplatin, docetaxel. FLOT-like: FLO = 5-FU, leukovorin, oxaliplatin or 5-FU ± trastuzumab ± pertuzumab; FOLFOX = 5-FU, leukovorin, oxaliplatin. ECF-like: ECX = epirubicin, cisplatin, capecitabin; EOF = epirubicin, oxaliplatin; and EOX = epirubicin, oxaliplatin, capecitabin; Others: pembrolizumab.

**Table 4:** Weight dynamics during different phases of the treatment. WL = weight loss, WG = weight gain.

	Median WL ^1^	Range ^2^
Neoadjuvant Phase	0%	WL 23%–WG 16%
Adjuvant Phase	4%	WL 24%–WG 8%
Whole Treatment	14%	WL 33%–WG 7%

^1^ Median WL indicates the median percentage of weight, compared to weight at time of diagnosis, that has been lost in that phase. ^2^ Range of weight changes in the cohort. It is expressed as percentage of weight at time of diagnosis that has been lost or gained in that phase.

**Table 5:** Cox regression analysis, OS.

Variable	Sig.	exp(B)	95% CI
Becker regression 1a/1b	0.008	0.068	0.009–0.503
Adjuvant chemotherapy performed	0.001	0.310	0.159–0.601
BMI < 25 kg/m2	0.162	1.595	0.829–3.071
WL ≥ 5% neoadjuvant	0.075	2.224	0.923–5.359
WL ≥ 14% whole treatment	0.140	1.744	0.833–3.653

**Table 6:** Cox regression analysis, DFS.

Variable	Sig.	exp(B)	95% CI
Becker regression 1a/1b	0.003	0.114	0.027–0.478
Adjuvant chemotherapy performed	0.006	0.415	0.222–0.776
BMI < 25 kg/m2	0.162	1.542	0.841–2.830
WL ≥ 5% neoadjuvant	0.176	1.810	0.766–4.273
WL ≥ 14% whole treatment	0.260	1.471	0.752–2.881

## Data Availability

Not applicable; anonymised data will be supplied upon request to the corresponding author.

## Supplementary Materials

The following supporting information can be downloaded at: https://www.mdpi.com/article/10.3390/curroncol29040221/s1, Table S1—Pts’ characteristics for male population only; Table S2—Characteristics of neoadjuvant and adjuvant chemotherapy for male population only; Table S3—List of the most used chemotherapy regimens, for male population only; Figure S1—Overall Survival and Disease Free Survival in the whole cohort; Figure S2—Overall Survival and Disease Free Survival for male population only; Figure S3—Kaplan-Meier curves for man population only according to different variables; Figure S4—Kaplan-Meier curves for male population only according to weight loss during the whole treatment; Figure S5—Kaplan-Meier curves for male population only according to weight loss during neoadjuvant therapy.
