# Supplementary material for: Prognostic Relevance of Weight and Weight Loss during Multimodal Therapy for Oesophagogastric Tumours
Source: Curr Oncol. 2022 Apr 12;29(4):2706–19. doi: 10.3390/curroncol29040221 (PMC9028417; doi:10.3390/curroncol29040221)
Supplement: Supplementary file 1 [file curroncol-29-00221-s001.zip › curroncol-1603347-supplementary.pdf]

Supplements

Table S1. Patients' characteristics for male population only.

Variable n = 108	Median	Range
Age	64	29-83
Weight	84 kg	55-118 kg
BMI	25.8 kg/m^2	16.5 – 39.4 kg/m^2
	Frequency	Percentage
Sex		
Males	108	100%
Localisation		
Oesophagus	65	60.2%
Stomach (incl. AEG III)	43	39.8%
Histology		
Adenocarcinoma	108	100%
Laurén Histology		
Intestinal Type	52	48%
Diffuse Type	28	25.9%
Mixed Type	9	8.3%
Not specified	19	17.6%
WHO-Histology		
Papillary	5	4.6%
Mucinous	6	5.6%
Tubular	24	22.2%
Signet Ring Cell	27	25%
Undifferentiated	8	7.4%
Not specified	38	35.2%

Table S2. Characteristics of neoadjuvant and adjuvant chemotherapy for male population only. Sum of percentages could be ≠100% because of rounding error.

Chemotherapy performed	Frequency	Percentage
Neoadjuvant	128	100%
Adjuvant	70	64.8%
**Nr. of cycles (neoadjuvant)**		
≤2	8	7.4%
3-4	98	90.7%
≥5	2	1.9%
**Nr. of cycles (adjuvant)**		
none	38	35.2%
≤2	16	14.9%
≥3	54	50%

Table S3. List of the most used chemotherapy regimens, for male population only. Sum of percentages could be ≠100% because of rounding error.

Regimens[1]	Frequency	Percentage
**Neoadjuvant (n= 128)**		
DCX	47	43.5%
ECF	34	31.5%
FLOT	18	16.7%
FLOT-like	5	4.6%
ECF-like	4	3.7%
**Adjuvant (n= 81)**		
DCX	35	50%
ECF	16	22.9%
FLOT	9	12.9%
FLOT-Like	6	8.5%
ECF-Like	3	4.3%
Others	1	1.4%

[1] DCX = Docetaxel, Cisplatin, Capecitabin; ECF = Epirubicin, Cisplatin, 5-FU; FLOT = 5-FU, Leukovorin, Oxaliplatin, Docetaxel, FLOT-Like: FLO = 5-FU, Leukovorin, Oxaliplatin; 5-FU ± Trastuzumab ± Pertuzumab; FOLFOX = 5-FU, Leukovorin, Oxaliplatin; ECF-Like: ECX = Epirubicin, Cisplatin, Capecitabin, EOF = Epirubicin, Oxaliplatin, EOX = Epirubicin, Oxaliplatin, Capecitabin; Others: Pembrolizumab.

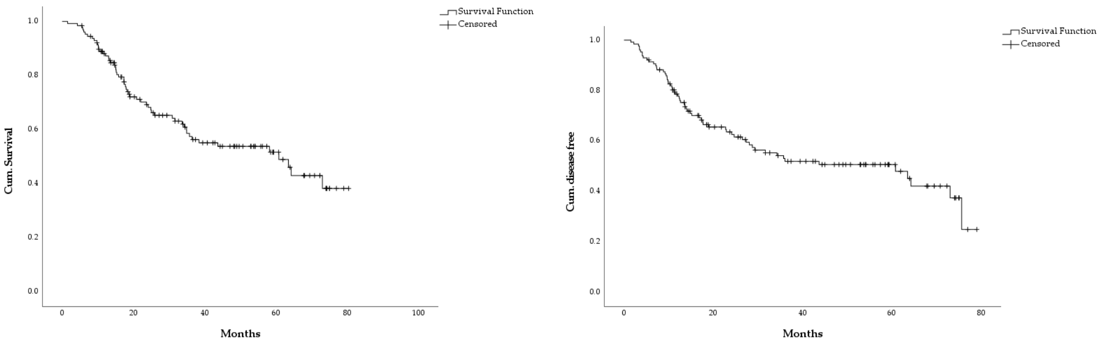

(**a**) (**b**)

Figure S1. Kaplan-Meier curves in the whole cohort. (**a**) Overall Survival (n=128); (**b**) Disease-Free Survival (n=128).

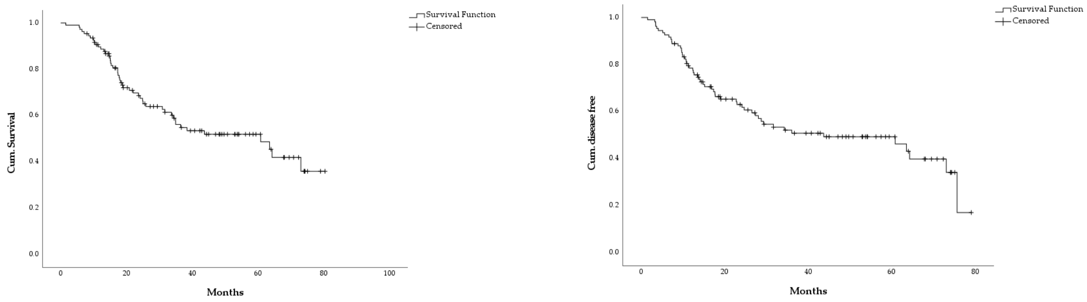

(**a**) (**b**)

Figure S2. Kaplan-Meier curves for male population only. (**a**) Overall Survival (n=108; 60.8 months; 95%-CI: 36.7 – 84.9); (**b**) Disease-Free Survival (n=108; 43.7 months, 95%-CI: 15.4 – 71.9).

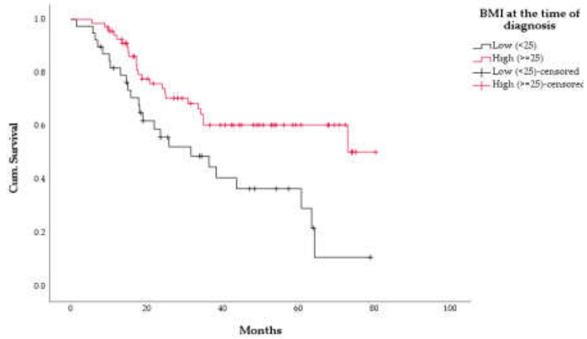

(**a**)

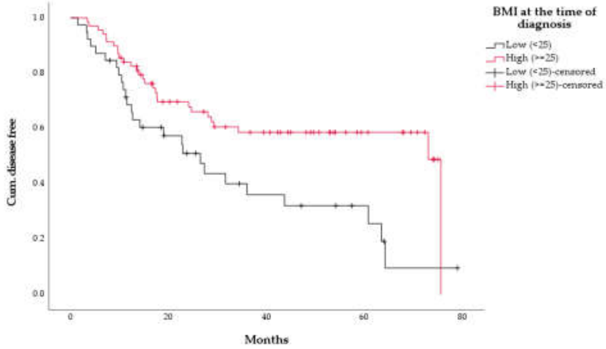

(**b**)

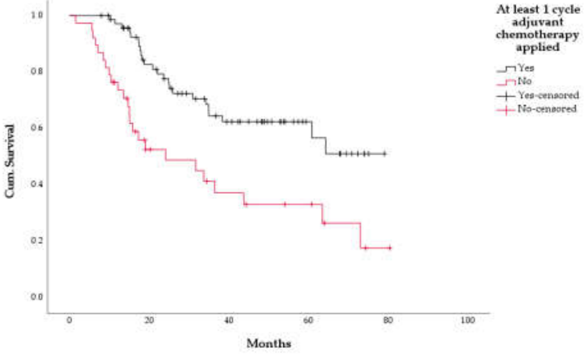

(**c**)

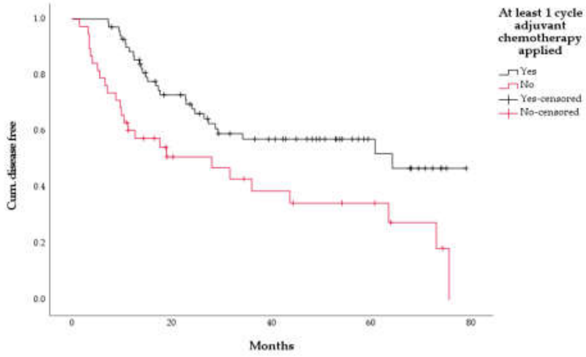

(**d**)

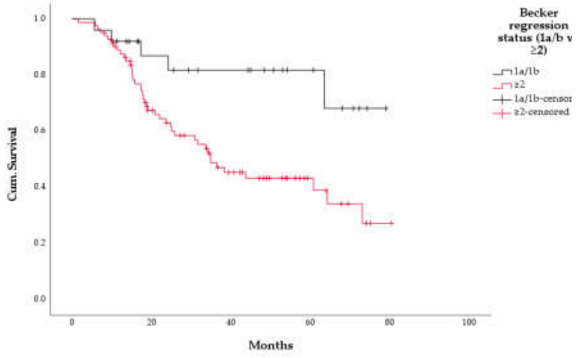

(**e**)

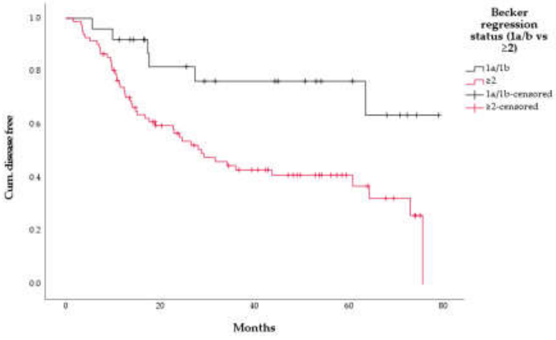

(**f**)

Figure S3. Kaplan-Meier curves for man population only according to different variables, as listed: (**a**) Overall Survival according to BMI (red line: BMI ≥25 kg/m2; n= 69; not reached; black line: BMI ≥25 kg/m2; n= 39; 31.7 months, 95%-CI: 12 – 51.3; p= 0.005); (**b**) Disease-Free Survival according to BMI (red line: BMI ≥25 kg/m2, n= 69; 73 months; 95%-CI 58.7 – 87.4; black line: BMI ≥25 kg/m2, n= 39; 26.5 months; 95%-CI: 16.2 – 36.8; p= 0.006); (**c**) Overall Survival according to application of adjuvant chemotherapy (black line: at least 1 cycle, n= 70; not reached; red line: no adjuvant chemotherapy, n= 38; 24.1 months; 95%-CI: 1.6 – 46.7; p= 0.000); (**d**) Disease-Free Survival according to application of adjuvant chemotherapy (black line:

at least 1 cycle, n= 70; not reached; red line: no adjuvant chemotherapy, n= 38; 11.3 months, 95%-CI: 5.8 – 50.3; p= 0.004); (**e**) Overall Survival according to Becker regression status, 107 analysable pts (black line: Becker regression status 1a/1b, n= 25; not reached; red line: Becker regression status ≥2, n= 82; 34.9 months, 95%-CI: 23.2 – 46.7; p= 0.01); (**f**) Disease-Free Survival according to Becker regression status, 107 analysable pts (black line: Becker regression status 1a/1b, n= 25; not reached; red line: Becker regression status ≥2, n= 82; 5.5 months; 95%-CI: 17.9 – 39.6; p= 0.005).

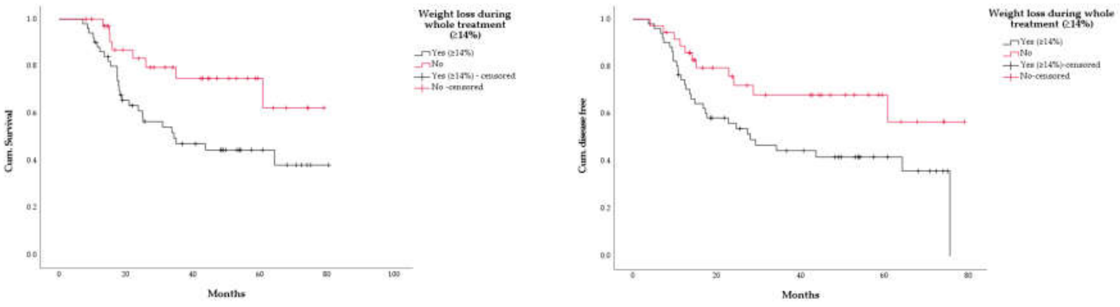

(**a**) (**b**)

Figure S4. Kaplan-Meier curves for male population only according to weight loss during the whole treatment (threshold: WL ≥14%, 87 analysable pts). (**a**) Overall Survival (black line: WL ≥14%, n= 51; 34.3 months, 95%-CI: 12 – 56.6; red line: WL <14%, n= 36; not reached; p= 0.017); (**b**) Disease-Free Survival (black line: WL ≥14%, n= 51; 28 months; 95%-CI: 14 – 42.1; red line: WL <14%, n= 36; not reached; p= 0.027).

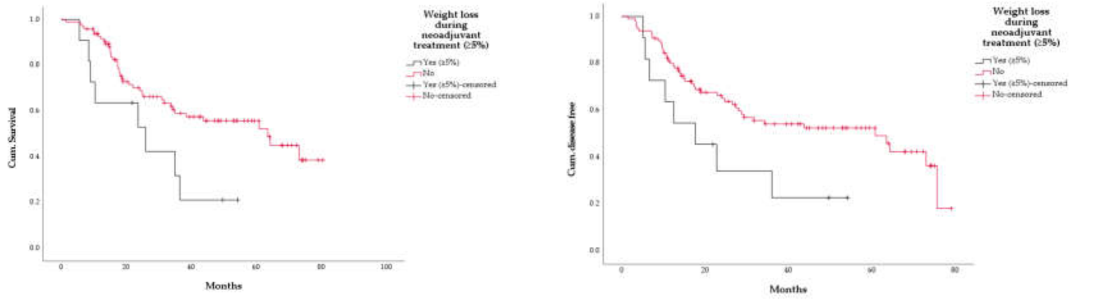

(**a**) (**b**)

Figure S5. Kaplan-Meier curves for male population only according to weight loss during neoadjuvant therapy (threshold: WL ≥5%, 108 analysable pts). (**a**) Overall Survival (black line: WL ≥5%, n= 11; 25.9 months; 95%-CI: 3.6 – 48.2; red line: WL <5%, n= 97; 63.5 months; 95%-CI: 40.8 – 86.2; p= 0.028); (**b**) Disease-Free Survival (black line: WL ≥5%, n= 11; 17.7 months; 95%-CI: 5.4 – 30; red line: WL <5%, n= 97; 60.8 months; 95%-CI: 31.3 – 90.3; p= 0.039).

^
